# “What About Automated Pain Recognition for Routine Clinical Use?” A Survey of Physicians and Nursing Staff on Expectations, Requirements, and Acceptance

**DOI:** 10.3389/fmed.2020.566278

**Published:** 2020-12-21

**Authors:** Steffen Walter, Sascha Gruss, Stephan Frisch, Joseph Liter, Lucia Jerg-Bretzke, Benedikt Zujalovic, Eberhard Barth

**Affiliations:** ^1^Medical Psychology Division, Department of Psychosomatic Medicine and Psychotherapy, University Hospital of Ulm, Ulm, Germany; ^2^Anaesthesiology Clinic, University Hospital of Ulm, Ulm, Germany

**Keywords:** automated pain recognition, artificial intelligence, acceptance, benefit, multimodal

## Abstract

**Background:** Over the last 12 years, the fundamentals of automated pain recognition using artificial intelligence (AI) algorithms have been investigated and optimized. The main target groups are patients with limited communicative abilities. To date, the extent to which anesthetists and nurses in intensive care units would benefit from an automated pain recognition system has not been investigated.

**Methods:** *N* = 102 clinical employees were interviewed. To this end, they were shown a video in which the visionary technology of automated pain recognition, its basis and goals are outlined. Subsequently, questions were asked about: (1) the potential benefit of an automated pain recognition in clinical context, (2) preferences with regard to the modality used (physiological, paralinguistic, video-based, multimodal), (3) the maximum willingness to invest, (4) preferences concerning the required pain recognition rate and finally (5) willingness to use automated pain recognition.

**Results:** The respondents expect the greatest benefit from an automated pain recognition system to be “to avoid over- or undersupply of analgesics in patients with limited communicative abilities,” a total of 50% of respondents indicated that they would use automated pain recognition technology, 32.4% replied with “perhaps” and 17.4% would not use it.

**Conclusion:** Automated pain recognition is, in principle, accepted by anesthetists and nursing staff as a possible new method, with expected benefits for patients with limited communicative skills. However, studies on automated pain recognition in a clinical environment and proof of its acceptance and practicability are absolutely necessary before such systems can be implemented.

## Introduction

To date, the recognition of pain in patients with reduced verbal communication skills has been realized by external observation only. However, all known external observation methods require professional expertise. Even if trained medical personnel could, in principle, record the intensity of pain several times a day or night using common observation instruments, continuous documentation is not possible. Automated pain recognition is a visionary means of utilizing valid and robust pain reaction patterns that can be recorded in a multimodal manner for a dynamic, high-resolution automated pain recognition system.

**Figure 1:**
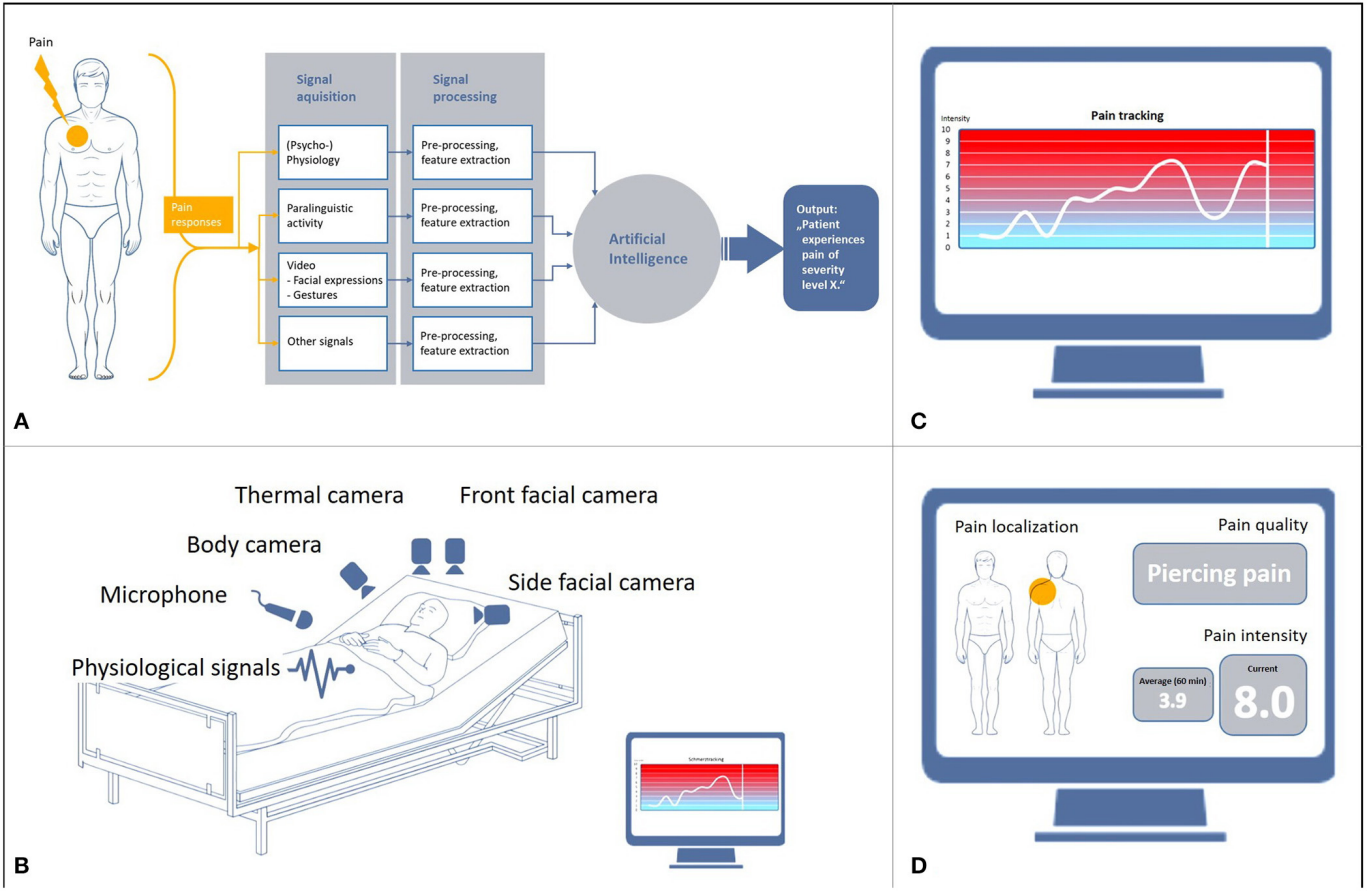
Basis and objectives of automated pain recognition. **(A)** Data processing in automated pain recognition, **(B)** Technical infrastructure of multimodality, **(C)** Monitoring of pain intensity, **(D)** Proposed monitoring of pain intensity, pain localization and quality.

The development of automated pain recognition (APR) was driven primarily by the finding that patients with limited communicative abilities are at a significant risk of being exposed to over- or undersupply of analgesics. In addition, external observation scales, which use a pain score based on sound expression, facial expression, body language, and physiological indicators, are only effective to a limited extent. In Germany, the BESD scale (1) is most commonly used for patients suffering from dementia. It mainly measures pain expression, vocalization, posture, and breathing. External observation is essentially based on the classification of pain components (2), which are referred to as surrogate markers and are used for the collocation of pain-associated stress. In order to further clarify the pain configuration, machine learning algorithms have been developed over the last 12 years and tested on healthy volunteers and patients with pain using a cross-validation approach. For an overview of the development of the APR method, see e.g., (3, 4). APR is an external observation method into which hardware and software components with artificial intelligence (AI) are integrated (Figures 1A,B). The aim of the research on classification algorithms of machine learning within AI is to optimize them with regard to robustness, i.e., to increase recognition rates and computing speed as well as preventing system crashes. Machine learning was initially applied in industrial settings for real-time classifiers in sorting plants (5), but is being used increasingly in medical areas (6) in which large data streams are generated. In general, machine learning is used to train an algorithm to distinguish categories or events (classes) and then use this algorithm to recognize categories or events in new or unknown data. Essentially, the focus of the development of the APR method in the field of pain research is currently on the recognition of pain intensity (Figure 1C) and on differentiation between the classes “baseline” vs. “pain threshold” vs. “pain tolerance.” Pain reactions are explicitly expressed in facial expressions (7, 8), gestures (9–11), paralinguistics (12, 13) and physiological reactions, which are measured with biosignal sensors (skin conductance/electrodermal activity [EDA], skin temperature, muscle activity [electromyography, EMG], heart rate variability [electrocardiogram, ECG]) (14–18). The first commercial unimodal (= using just one signal) prototypes were developed on this basis (19). The term “information fusion” (Figure 1A) is used to describe cases where there is a “fusion” of multiple signals (= multimodal). A number of studies have shown that detection rates resulting from multimodal fusions (20, 21) were significantly higher (22, 23) than unimodal detection rates. The initial transfers from the basic research on multimodal pain intensity recognition into clinical applications are in progress.

Nevertheless, the extent to which the detection of pain intensity can be differentiated from other states of non-pain-related stress in a specific context (e.g., delirium, panic, shortness of breath, etc.) is also of high relevance. This requires further intensive and fundamental research through laboratory experiments. A further objective is the development of an APR system for the recognition of pain quality (pulling, stabbing, drilling) and pain localization (3, 4).

The present study is intended to answer the following questions for the first time:

1. Do physicians and nurses see a benefit in the clinical use of APR?
2. Which modality (unimodal or multimodal) (Figure 1A) would they favor?
3. How much would they be willing to invest financially in APR technology?
4. What pain detection rate would they consider clinically sufficient?
5. Would physicians and nurses actually use APR in everyday hospital life?

The Clinic of Anesthesiology at University Hospital Ulm was chosen for the monocentric survey.

## Methods

### Respondents and Response Rate

In total, 179 employees of the Department of Anesthesiology at University Hospital Ulm were asked to take part in the survey. Of these, *N* = 102 (56.9% response rate) employees were willing to participate in the survey (men = 48, women = 54; average age 40.31 years [SD = 11.5]). In detail, 89 physicians (senior physicians and assistants) of the Clinic of Anesthesiology in Ulm were contacted by e-mail and asked to participate in the study. A total of 43 physicians (48.3% response rate) participated in the survey. In addition, 90 nurses working in the intensive care units CGI and CF1 (https://www.uniklinik-ulm.de/anaesthesiologie/interdisziplinaere-operative-intensivmedizin-ioi.html) were personally visited by an assistant and asked to complete the survey using a tablet. A total of 59 nurses (65.6% response rate) were willing to answer the questions.

### Questionnaire

**Figure 2:**
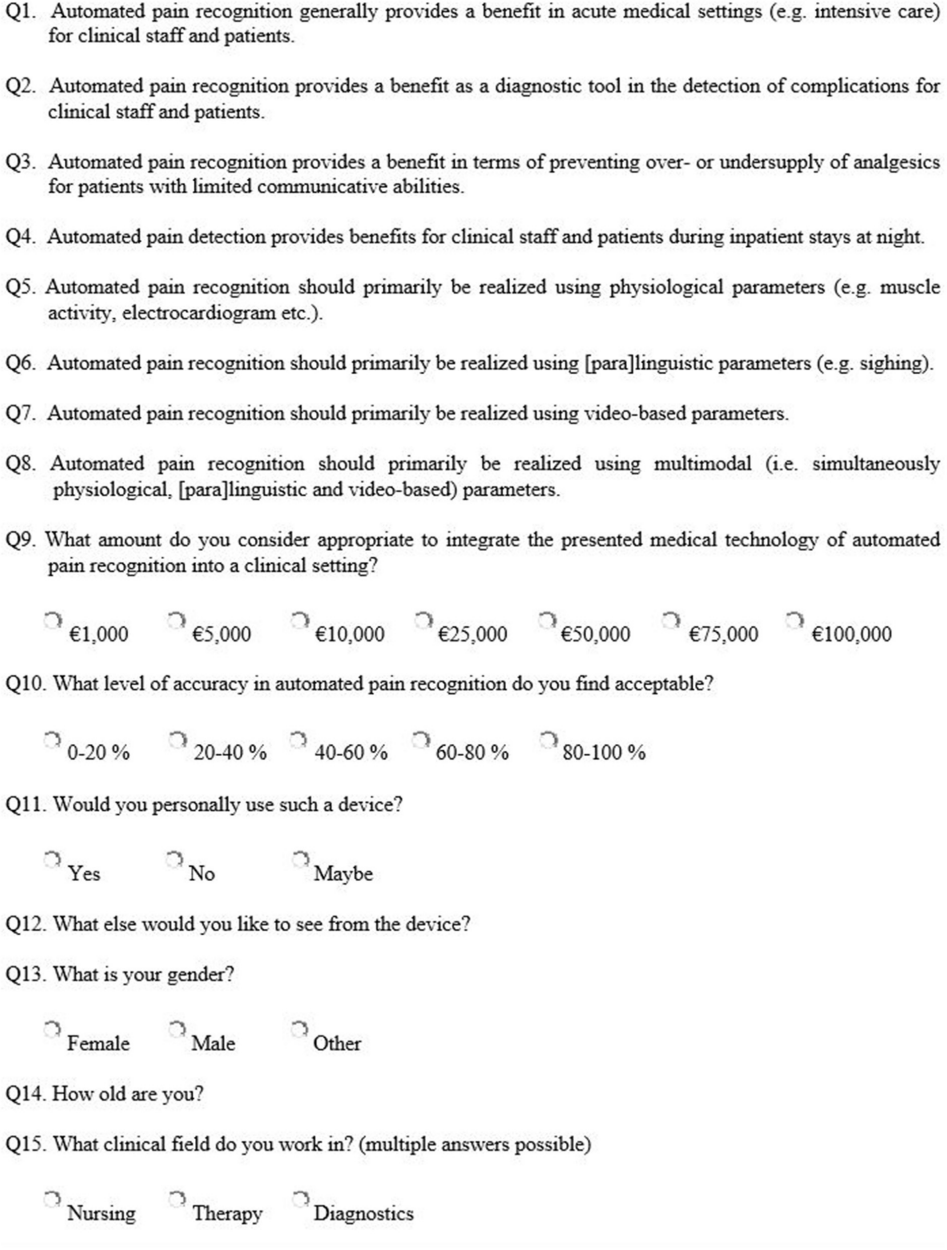
Questionnaire on the acceptance of automated pain recognition.

All participants had to watch a specially produced video of an automated pain recognition system (https://youtu.be/kKwEGa-oV5s), which showed the vision of APR using AI algorithms AI (Figure 1). Afterwards, a short questionnaire with 15 questions (Q1–Q15) was presented, 8 of these (Q1–Q8) were Likert scale questions (ranging from “*1*” = “*not at all*” to “*6*” = “*absolutely*”) (Figure 2). Participants were free to ask questions, but no questions were asked about the content.

### Statistical Analyses

For questions 1–8, mean values were calculated in each case and shown in histograms. The results are not normally distributed. In addition, a Friedman test (with a corresponding pair comparison test) was performed with regard to the clinical benefit (Q1–Q4) to identify any significant differences in the questions concerning the benefits of the system. A Friedman test was also performed with regard to the preferred modality (physiological, paralinguistic, video-based or multimodal) (Q5–Q8). Relative frequencies were determined for Q10–Q15 (except for Q12). Q12 was only answered by 10 subjects and was not qualitatively evaluated in the present study.

The SPSS 25 statistical software was used to calculate all statistical analyses.

## Results

**Figure 3:**
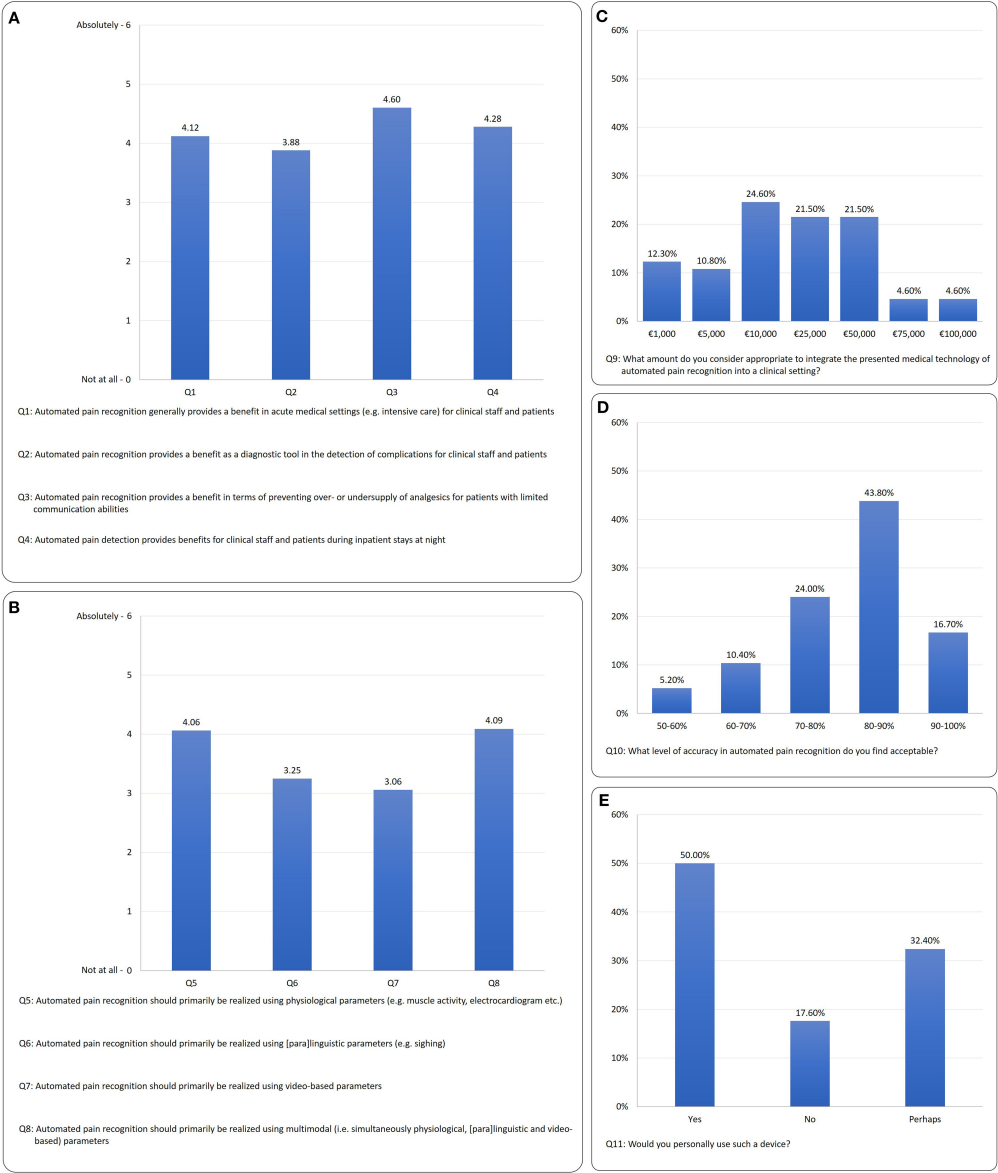
Survey results: **(A)** context of clinical benefit (Q1–Q4), **(B)** preferred modality (Q5–Q8), **(C)** maximum financial investment (F9), **(D)** required detection rate (Q10), **(E)** willingness to use APR (Q11).

The results are shown in Figure 3 (3A: benefits of APR in clinical context, 3B: preferred modality (physiological, paralinguistic, video-based, multimodal), 3C: maximum financial investment, 3D: required pain detection rate and 3E: willingness to use APR.

For the questions on the benefits of APR (Q1–Q4), the differentiation shows a significant preference among the benefits expected *X*^2^ = *14.15*; *p* ≤ *0.001*; *N* = *102*. The greatest benefit expected is that “*Automated pain recognition provides a benefit in terms of preventing over- or undersupply of analgesics for patients with limited communicative abilities*” (Q3) with *M* = *4.60, SD* = *1.5*. In a *post-hoc* (Wilcoxon) comparison, Q3 receives significantly more positive answers than:

- Q1 “*Automated pain recognition generally provides a benefit in acute medical settings (e.g., intensive care) for clinical staff and patients*” (*Z* = −4.33, *p* = 0.001),
- Q2 “*Automated pain recognition provides a benefit as a diagnostic tool in the detection of complications for clinical staff and patients*” (*Z* = −5.22, *p* = 0.001) and
- Q4 “*Automated pain detection provides benefits for clinical staff and patients during inpatient stays at night*” (*Z* = −2.52, *p* = 0.012).

With regard to the questions on the preferred modality of APR system (Q5–Q8), a clear preference was apparent *X*^2^ = *68.89, p* ≤ *0.001, N* = *102*. The results showed that multimodality (Q8) *M* = *4.09*; *SD* = *1.87*, similarly to the physiological modality (Q5) *M* = *4.06*; *SD* = *1.43*, was favored by the majority of respondents. The *post-hoc* (Wilcoxon) tests for the physiological modality (Q5) vs.:

- the paralinguistic modality (Q6) (*Z* = −5.16, *p* = 0.001) and
- the video-based modality (facial expression/gesture) (Q7) (*Z* = −5.66, *p* = 0.001)

are significant.

Likewise, the *post-hoc* (Wilcoxon) tests for multimodality (Q8) vs.:

- the paralinguistic modality (Q6) (*Z* = −4.35, *p* = 0.001) and
- the video-based modality (facial expression/gesture) (Q7) (*Z* = −6.03, *p* = 0.001)

are also significant.

Furthermore, additional explorative tests were carried out for detecting differences in gender (Q13), age (Q14), profession (Q15) and decision use APR (Q11) regarding Q1–Q8 (see Supplementary Table):

There was no significant difference between men and women. For questions Q1, Q3, Q4, Q7, Q8, higher values are reported significant for the “younger” respondents. Physicians have rated Q8 significantly higher. Q1–Q8 were rated lower for respondents who would decide against APR (Q11, “no”).

Figure 3C shows that the majority of respondents consider a sum of €10,000 to €50,000 appropriate to invest in an APR prototype.

Figure 3D shows that the majority of clinicians would accept a pain detection accuracy of >80%.

In terms of willingness to use the device, Figure 3E shows that 50% of respondents would use it; 32.4% “perhaps” and 17.6% would not use the technology. The age effect can also be found here: 55.8% of the younger group would use it; 36.5% “perhaps” and 7.7% would not use the technology. In terms of the older participants, 40.4% of respondents would use it; 29.8% “perhaps” and 29.8% would not use the technology.

The following answers were given regarding Q12 (“*What else would you like to see from the device?”*):

- suitable for all ages
- pain recognition level 1–10, automatic alert if pain level >5
- user-friendly & simple handling/operation, self-explanatory, easy to setup (<2 min), easy to clean
- simple visualization
- small & compact design, wireless or max. one cable
- out of reach of patient, should not restrict patients
- reliable (measurement)
- if pain detected then recommendation for action or adapted administration of medication
- (modular) design for integration into existing monitoring systems
- possibility to print measured parameters, integration in electronic medical record
- input/consideration of possible error sources such as hyperactive delirium etc.

“Reliability” and “usability” were mentioned most frequently.

## Discussion

The development of an APR methodology has advanced in an experimental setting; uni- and multimodal pain intensity recognition rates of >80% have already been achieved with machine learning procedures [overview e.g., in (3, 4)]. The next step will be the development of a prototype to test the automated recognition of pain intensity in clinical practice, which ultimately requires the acquisition of large data sets (big data). In order to achieve this goal, it is first necessary to gain an impression of the expectations and requirements among physicians and nursing staff with respect to APR and their potential willingness to use APR technology in clinical practice.

Ultimately, the acceptance of APR in everyday clinical practice depends on factors such as data protection, user-friendliness, miniaturization of hardware, etc. Another important factor is how patients – with and without limited communicative skills — will tolerate and accept pain detection using a camera, microphone and multiple contact sensors, and also the extent to which they would consent to the continuous collection and processing of their data, as required for this method.

The fact that ultimately 50% of respondents have a positive attitude toward APR, 30.4% would “perhaps” and 17.6% would not use this technology, indicates that APR research and development should continue constructively and critically. It is important to bear in mind that there is an age effect. At 29.8%, older participants (>40 years) are rather critical to APR. It can also be seen that participants who would not use APR (Q11), in principle have less affinity for the area of application (Q1–Q4) and modality preferences (Q5–Q8). Further studies should help to clarify the reasons why a small group of physicians and nurses reject APR and why they are rather critical to the different areas of applications (Q1–Q4) and modalities (Q5–Q8). However, there is a tendency to assume that the aspects of quality criteria (reliability, validity) and user-friendliness will be important, as these were mentioned most frequently in the expectations (Q12). The aspect of user friendliness in particular could play a greater role for the “older” group.

The authors are not aware of any other studies on the acceptance of APR in the clinical setting.

A recent study from China (24) indicates an acceptance rate of 99% for the use of AI in medicine among 2,780 physicians and non-medical staff (where 54.3% of respondents were physicians). The higher general acceptance found in this study compared to our survey likely also reflects cultural differences between China and Germany. In total, 92.7% of the physicians and 81.7% of the non-medical staff we surveyed recognize the necessity of using AI to support diagnostics and therapy, while 60.1% of the medical staff believe that AI should be used to improve diagnoses. An increase in diagnostic accuracy through AI is expected by as many as 73.4% of non-medical personnel.

In particular, the present survey shows that physicians and nursing staff in an anesthesiological intensive care unit would see the benefit of APR in the prevention of over- or undersupply of analgesics in patients with impaired communication skills. Thus, the potential of APR is clearly seen to lie in improving the therapeutic care of these patients in intensive care units in terms of pain management and to reduce suffering and side effects. This confirms the appropriateness of the adopted focus of the development of APR on patients with impaired communicative skills, which should therefore be pursued and not reconsidered.

It is not surprising that, with regard to APR, physicians and nurses in an anesthesiological intensive care unit would give the highest priority to a unimodal method with a physiological modality, as they are traditionally familiar with this modality. On the other hand, a multimodal APR would also be given clear priority, so the complex multimodal approach should also be tested in a clinical setting. Facial and gestural reactions could possibly be recorded entirely by means of EMG surface sensors (=physiological). Nevertheless, the differentiation of pain from the behaviors in states such as of delirium, panic or breathing difficulties is only possible to a very limited extent using “purely” physiological means.

The majority of respondents would consider a sum of at least €10,000 appropriate to invest in APR technology. From the authors' point of view, it seems realistic that an APR device could be purchased for this amount of money once the development of the method has been completed. There is an indication that the mass production of APR devices would make it possible to further reduce costs. In other words, a larger intensive care unit (20–40 patients) would have to invest about €200,000 to €400,000 in order to equip all of its beds with this technology. However, it would also be conceivable to equip a small number of beds especially for patients with limited communicative abilities.

The respondents indicate that they would accept recognition rates of about 80% or higher, which is comparable to recognition rates in other areas of affective computing (25).

Limitation: Forty-three and one tenths percentage of the staff were not willing to participate in the survey, the reasons being exclusively, a workload in the intensive care unit.

The authors are planning to pilot APR prototype in an intensive care unit as part of a future study. Furthermore, they plan to conduct an international multicentric acceptance survey on automated pain recognition.

In the future, multimodal data sets should also be collected in clinical acute medicine settings in order to test and further develop APR and AI algorithms.

## Data Availability Statement

The datasets presented in this article are not readily available due to German data protection regulations. Requests to access the datasets should be directed to Steffen Walter, steffen.walter@uni-ulm.de.

## Ethics Statement

The studies involving human participants were reviewed and approved by Ethics Committee Ulm. Written informed consent for participation was not required for this study in accordance with the national legislation and the institutional requirements.

## Author Contributions

SW: literature search, data analysis, and writing. SG: study design and writing. SF: critical revision and writing. JL: data collection. LJ-B and BZ: critical revision. EB: critical revision and data interpretation. All authors contributed to the article and approved the submitted version.

## Conflict of Interest

The authors declare that the research was conducted in the absence of any commercial or financial relationships that could be construed as a potential conflict of interest.

## Abbreviations

APR: automated pain recognition
AI: artificial intelligence
EMG: electromyography
EDA: electrodermal activity
ECG: electrocardiogram
Q: question.
