# Supplementary material for: “What About Automated Pain Recognition for Routine Clinical Use?” A Survey of Physicians and Nursing Staff on Expectations, Requirements, and Acceptance
Source: Front Med (Lausanne). 2020 Dec 21;7:566278. doi: 10.3389/fmed.2020.566278 (PMC7779395; doi:10.3389/fmed.2020.566278)
Supplement: Supplementary file 1 [file Table_1.docx]

Supplementary: Comparison gender (Q13), age (younger: ≤ 40 yrs., older: > 40 yrs.; age for three participants is missing) (Q14), profession (Q15), decision for APR (Q11). Independent testing with Mann-Whitney-U-Test and Kruskal-Wallis-Test (post hoc)

| **variables** | **conditions** | **Q1** | | | | **Q2** | | | | **Q3** | | | | **Q4** | | | |
| --- | --- | --- | --- | --- | --- | --- | --- | --- | --- | --- | --- | --- | --- | --- | --- | --- | --- |
|  |  | *M* | *SD* | *Z/H* | *p* | *M* | *SD* | *Z/H* | *p* | *M* | *SD* | *Z/H* | *p* | *M* | *SD* | *Z/H* | *p* |
| gender (Q13) | male (N = 48) | 4.08 | 1.38 | -.510^1^ | .608 | 4.08 | 1.37 | -1.16^1^ | .245 | 4.60 | 1.33 | -.38^1^ | .704 | 4.40 | 1.47 | -.57^1^ | .569 |
|  | female (N = 54) | 4.15 | 1.64 |  |  | 3.7 | 1.62 |  |  | 4.59 | 1.56 |  |  | 4.19 | 1.61 |  |  |
| age (Q14) | younger (N = 52) | 4.56 | 1.24 | -3.03^1^ | **.002** | 4.13 | 1.28 | -1.65^1^ | .099 | 4.88 | 1.31 | -2.16^1^ | **.031** | 4.60 | 1.29 | -2.02^1^ | **.043** |
|  | older (N = 47) | 3.57 | 1.67 |  |  | 3.55 | 1.72 |  |  | 4.26 | 1.58 |  |  | 3.87 | 1.74 |  |  |
| profession (Q15) | physicians (N = 43) | 4.35 | 1.43 | -1.37^1^ | .169 | 3.98 | 1.42 | -.445^1^ | .656 | 4.79 | 1.32 | -.970^1^ | .332 | 4.56 | 1.44 | -1.60^1^ | .110 |
|  | nurses (N = 59) | 3.95 | 1.57 |  |  | 3.81 | 1.58 |  |  | 4.46 | 1.54 |  |  | 4.08 | 1.6 |  |  |
| decision for APR  (Q11) | yes (N = 51) | 5 | .894 | 44.2^2^ | .**001** | 4.51 | 1.12 | 24.88^2^ | **.001** | 5.27 | .918 | 31.34^2^ | **.001** | 5.00 | .98 | 27.73^2^ | **.001** |
|  | maybe (N = 33) | 3.68 | 1.49 |  |  | 3.71 | 1.51 |  |  | 4.29 | 1.49 |  |  | 3.97 | 1.59 |  |  |
|  | no (N = 18) | 2.33 | 1.09 |  |  | 2.39 | 1.38 |  |  | 3.17 | 1.47 |  |  | 2.78 | 1.56 |  |  |
| decision for APR  post-hoc (Q11) | no-yes | - | - | 6.33 | **.001** | - | - | 4.93 | **.001** | - | - | 5.37 | **.001** | - | - | 5.06 | **.001** |
|  | no-maybe | - | - | -2.92 | **.004** | - | - | -2.88 | **.004** | - | - | -2.56 | **.032** | - | - | -2.46 | **.042** |
|  | maybe-yes | - | - | 3.95 | **.001** | - | - | 2.28 | **.023** | - | - | 3.23 | **.004** | - | - | 2.99 | **.008** |
| **variables** | **conditions** | **Q5** | | | | **Q6** | | | | **Q7** | | | | **Q8** | | | |
|  |  | *M* | *SD* | *Z/H* | *p* | *M* | *SD* | *Z/H* | *p* | *M* | *SD* | *Z/H* | *p* | *M* | *SD* | *Z/H* | *SD* |
| gender (Q13) | male (N = 48) | 4.10 | 1.29 | **-**.010^1^ | .992 | 3.17 | 1.17 | -.69^1^ | .488 | 3.23 | 1.28 | -1.16^1^ | .246 | 4.46 | 1.5 | -1.67^1^ | .096 |
|  | female (N = 54) | 4.02 | 1.55 |  |  | 3.21 | 1.50 |  |  | 2.91 | 1.47 |  |  | 3.76 | 1.94 |  |  |
| age (Q14) | younger (N = 52) | 4.15 | 1.21 | **-.**230^1^ | .818 | 3.40 | 1.27 | -1.38^1^ | .169 | 3.44 | 1.26 | -2.89^1^ | **.004** | 4.65 | 1.47 | -2.94^1^ | .**003** |
|  | older (N = 47) | 3.94 | 1.65 |  |  | 3.09 | 1.47 |  |  | 2.66 | 1.45 |  |  | 3.53 | 1.90 |  |  |
| profession (Q15) | physicians (N = 43) | 3.84 | 1.29 | -1.57^1^ | .117 | 3.26 | 1.22 | -.076^1^ | .939 | 3.26 | 1.20 | -1.29^1^ | .197 | 5.02 | 1.37 | -4.80^1^ | **.001** |
|  | nurses (N = 59) | 4.22 | 1.51 |  |  | 3.24 | 1.45 |  |  | 2.92 | 1.52 |  |  | 3.41 | 1.73 |  |  |
| decision for APR  (Q11) | yes (N = 51) | 4.22 | 1.19 | 7.49^2^ | **.024** | 3.51 | 1.14 | 9.43^2^ | **.009** | 3.35 | 1.31 | 18.23^2^ | **.001** | 4.75 | 1.48 | 22.72^2^ | **.001** |
|  | maybe (N = 33) | 4.32 | 1.39 |  |  | 3.26 | 1.56 |  |  | 3.35 | 1.37 |  |  | 3.97 | 1.77 |  |  |
|  | no (N = 18) | 3.11 | 1.75 |  |  | 2.33 | 1.24 |  |  | 1.78 | 1 |  |  | 2.5 | 1.5 |  |  |
| decision for APR  post-hoc (Q11) | no-yes | - | - | 2.3 | **.022** | - | - | 3.03 | .**003** | - | - | 4.05 | .**001** | - | - | 4.69 | **.001** |
|  | no-maybe | - | - | -2.66 | **.008** | - | - | -2.49 | **.013** | - | - | -3.78 | **.001** | - | - | -2.62 | **.009** |
|  | maybe-yes | - | - | .67 | .500 | - | - | .447 | .655 | - | - | .017 | .986 | - | - | 2.32 | **.021** |

Significant results are bold.

^1^: Z = Mann-Whitney-U-Test

^2^: H = Kruskal-Wallis-Test (decision for technology)
